# High school music classes enhance the neural processing of speech

**DOI:** 10.3389/fpsyg.2013.00855

**Published:** 2013-12-06

**Authors:** Adam Tierney, Jennifer Krizman, Erika Skoe, Kathleen Johnston, Nina Kraus

**Affiliations:** ^1^Auditory Neuroscience Laboratory, Northwestern UniversityEvanston, IL, USA; ^2^Department of Communication Sciences, Northwestern UniversityEvanston, IL, USA; ^3^Bilingualism and Psycholinguistics Research Group, Northwestern UniversityEvanston, IL, USA; ^4^Walter Payton College Preparatory High SchoolChicago, IL, USA; ^5^Institute for Neuroscience, Northwestern UniversityEvanston, IL, USA; ^6^Department of Neurobiology and Physiology, Northwestern UniversityEvanston, IL, USA; ^7^Department of Otolaryngology, Northwestern UniversityEvanston, IL, USA

**Keywords:** hearing, training, music, brainstem, auditory perception

## Abstract

Should music be a priority in public education? One argument for teaching music in school is that private music instruction relates to enhanced language abilities and neural function. However, the directionality of this relationship is unclear and it is unknown whether school-based music training can produce these enhancements. Here we show that 2 years of group music classes in high school enhance the neural encoding of speech. To tease apart the relationships between music and neural function, we tested high school students participating in either music or fitness-based training. These groups were matched at the onset of training on neural timing, reading ability, and IQ. Auditory brainstem responses were collected to a synthesized speech sound presented in background noise. After 2 years of training, the neural responses of the music training group were earlier than at pre-training, while the neural timing of students in the fitness training group was unchanged. These results represent the strongest evidence to date that in-school music education can cause enhanced speech encoding. The neural benefits of musical training are, therefore, not limited to expensive private instruction early in childhood but can be elicited by cost-effective group instruction during adolescence.

## INTRODUCTION

The role of music education in schools is under debate, as music competes with other in-school programs for access to a small pool of funding. At the center of the debate is the question of whether in-school music education bolsters the development of the brain and mind. It has been hypothesized that music can function as a training ground for language skills (Patel, 2011) as a result of its acoustic and structural overlap with language and its tendency to capture attention and emotion (Menon and Levitin, 2005). In support of this hypothesis, private, one-on-one music instruction improves language abilities including verbal memory (Chan et al., 1998), literacy (Tallal and Gaab, 2006; Moreno et al., 2009), verbal intelligence (Forgeard et al., 2008; Moreno et al., 2011), and speech processing (Kolinsky et al., 2009; François et al., 2012). Across the lifespan, highly trained musicians also display an impressive advantage for perceiving speech in background noise relative to their musically naive counterparts (Parbery-Clark et al., 2009b, 2011; Strait et al., 2012; Zendel and Alain, 2012). Linked to this behavioral advantage is a greater neural resilience to background noise and other forms of acoustic degradations (Bidelman and Krishnan, 2010). Noise delays the neural response to sound (Burkard and Sims, 2002); however, faster neural responses to degraded speech are consistently linked to music training (Parbery-Clark et al., 2009a; Kraus and Chandrasekaran, 2010; Strait et al., 2012), enhanced speech-in-noise perception (Parbery-Clark et al., 2009a), and better reading abilities (Anderson et al., 2010) across the lifespan (see Kraus and Chandrasekaran, 2010; Strait and Kraus, 2013 for reviews).

There is converging evidence, therefore, that music training can improve neural encoding of speech. An alternate explanation, however, is that musicians have inherently advanced auditory skills and are thus drawn to musical training. Longitudinal work investigating both a musical training and a control training group can conclusively show that musical training produces speech encoding benefits and rule out pre-existing differences in neural function. Longitudinal studies have revealed that music training can lead to enhanced auditory neural function (Fujioka et al., 2006; Shahin et al., 2008; Moreno et al., 2009; Chobert et al., 2012; François et al., 2012; Strait et al., 2013). However, the training used in these studies was either computerized or one-on-one music lessons, and it is unclear whether group music lessons within a school setting yield similar outcomes. The investigation of the neural effects of in-school music training, therefore, is crucial for providing empirical evidence relevant to the debate about the efficacy of music education in schools. Our study was unique in that it accessed adolescents undergoing group music classes within a public school setting.

Our study was further motivated by the fact that public music education is on the decline (National Endowment for the Arts survey, Rabkin and Hedberg, 2011) and that private music lessons, due to their expense, are more accessible to socioeconomically advantaged, relative to disadvantaged, families (Duke et al., 1997). By partnering with schools that offer music education to low-income minority communities, we hoped to understand the extent to which in-school musical training might benefit a population that otherwise might not have access to music education.

**FIGURE 1:**
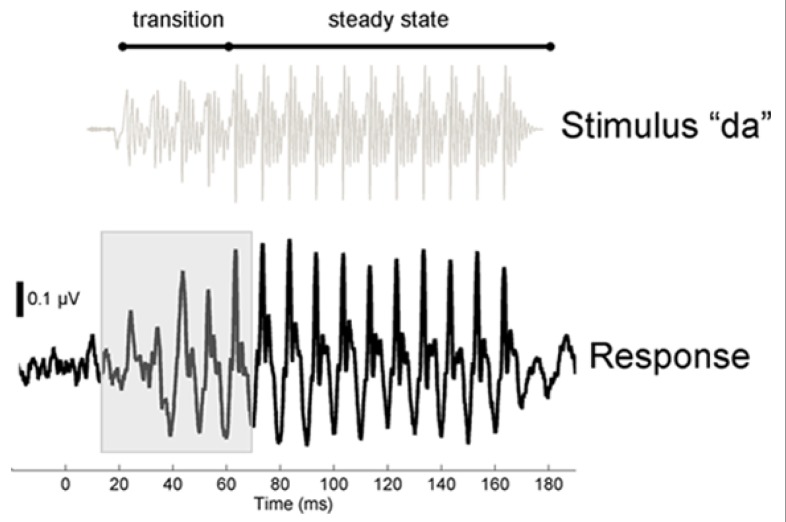
**Stimulus and response time-domain waveforms.** To illustrate the temporal characteristics of the stimulus and auditory brainstem response, the baseline grand average response (average of trained and control group) is plotted below the waveform for the stimulus [da], which was presented in a noisy background of multi-talker babble. The speech stimulus is divided acoustically into a transition region, during which the speech formants change linearly as the sound moves from the consonant to the vowel, and a steady-state vocalic portion of the stimulus, where the spectrotemporal profile of the stimulus is stable. Compared to the steady-state region, the formant transition region is more strongly masked by the presence of noise. The response to the spectrotemporally dynamic transition is highlighted (10–70 ms). We compared the stimulus and the response by calculating the time lag, for each subject, at which the two waveforms align more closely. For the grand average waveform plotted here, the maximum correlation is achieved at a lag of 7.9 ms. Consequently, for this graph the stimulus waveform was shifted by 7.9 ms to the right to maximize the visual alignment with the response.

Using a longitudinal design, we investigated how in-school music training affects the adolescent brain by studying high school students from the Chicago Public School district. As students from a district serving largely socioeconomically disadvantaged families, these subjects represent a population that has been under-studied by biological scientists. Participants were tested prior to and immediately following 2 years of training. We hypothesized that classroom musical instruction increases the brain’s resilience to background noise and we, therefore, predicted that after training, music students would have earlier neural responses to speech presented in noise. Electrophysiological responses were measured to a synthesized speech syllable presented repetitively in the presence of background noise (six-talker babble; **Figure 1**; Skoe and Kraus, 2010). Analyses focused on the neural response to the dynamically changing portion of the syllable (10–70 ms), as earlier timing within this response region has been linked with musical training (Parbery-Clark et al., 2012).

## MATERIALS AND METHODS

### PARTICIPANT AND GROUP CHARACTERISTICS

Subjects were 43 adolescents attending three public high schools in Chicago [music training *n* = 21 (11 female), fitness training *n* = 22 (7 female, sex difference: *p* > 0.15, chi-square = 1.87)]. 14 students participated in the study at pre-test but were unable to come back for the post-testing phase. Four of these students dropped out of the study voluntarily and 10 were unable to return for personal reasons (school transfers, family emergencies, medical conditions, etc.) Mean age at pretest was 14.6 years (standard deviation 0.46) for the music training group and 14.7 (0.34) for the fitness training group. This age difference was not significant according to an independent *t*-test, *t*(41) = 0.49, *p* > 0.5. As part of the curriculum for each of these schools, all students must enroll (for credit) in either music or Junior Reserve Officer’s Training Corp (JROTC) classes which meet 2–3 times each week, averaging about 3 h of instruction each week. Music students participated in either band (*n* = 9) or choral (*n* = 12) class.

Students were tested prior to beginning music or fitness classes, providing a baseline measure of neural function. This baseline measure was critical in establishing that differences in brain function following 2 years of training are linked to training and not confounded by initial group differences. While no subjects in the fitness training group had any prior musical training, two subjects in the musical training group had a small amount of formal musical training for 1 and 6 years. However, given that the two groups were matched on neural timing at pre-test, we interpret any differences in year-to-year changes in neural timing to the different training regimens that the two groups received during the study. Groups were matched on pre-training performance using measures of IQ (Wechsler Abbreviated Scale of Intelligence, WASI; musicians: 99.57 ± 11.57; ROTC: 98.23 ± 7.45; *F* = 0.207, *p* = 0.651), reading abilities (Word Attack, Woodcock-Johnson III test battery; musicians: 97.62 ± 9.68; ROTC: 101.68 ± 11.76; *F* = 1.521, *p* = 0.225), and auditory working memory (Auditory Working Memory, Woodcock-Johnson III test battery; musicians: 103.71 ± 11.03; ROTC: 103.64 ± 10.46; *F* = 0.001, *p* = 0.981). Groups were matched on SES using maternal education as an index of SES (Hackman et al., 2010; Kolmogorov–Smirnov *z* = 0.986, *p* = 0.285). Both groups were from predominately low SES backgrounds, with the majority of subjects reporting a maternal education level of high school graduate. Additional inclusionary criteria were normal hearing as determined by air conduction thresholds (<20 dB normal hearing level for octaves from 125 to 8000 Hz), click-evoked brainstem response latencies within normal limits (5.41–5.97 ms; the 100-μs rarefaction click stimulus was presented at 80 dB sound pressure level (SPL) at a rate of 31/s), and no external diagnosis of a reading disorder.

### DESCRIPTION OF MUSIC CURRICULUM

The curriculum is designed as a 4 year sequence that takes incoming students at a beginning level and prepares them to participate in college-level music classes. Band and choir curricula are developed in tandem so that students in either track graduate from high school with a similar level of musical skill. Singers receive additional keyboard training. Students participate in a minimum of two public performances per year. Lessons include practice in sight reading, singing/playing technique, and regular assessments to measure student progress. Assessments include written exams related to music theory, singing/playing exams that address continuous growth as well as concert readiness, and content-based writing assignments.

### DESCRIPTION OF FITNESS CURRICULUM

This curriculum is also designed as a 4 year sequence. Its primary focus is to develop leadership skills, strengthen character, and instill self-discipline through classroom instruction and fitness training. Students are graded and promoted based on demonstrating knowledge and mastery of the concepts covered in the classroom as well as achieving muscular and cardiovascular fitness milestones.

### STIMULUS AND RECORDING

Stimulus and recording parameters followed those described in Skoe and Kraus (2010). The stimulus was the synthesized speech syllable [da], a six-formant, 170 ms sound characterized by an initial stop burst followed by a 40 ms voiced formant transition. The transition is followed by a 120 ms steady-state [a] vowel in which the formants are unchanging. The [da] stimulus was presented in alternating stimulus polarities at a rate of 3.98/s to the right ear at 80 dB SPL through an insert earphone (ER-3; Etymotic Research) using the stimulus presentation software NeuroScan Stim2 (Compumedics). Ag/Ag-Cl electrodes were applied in a vertical montage from Cz to right earlobe with forehead as ground. Responses were recorded in a sound-attenuated, electrically shielded chamber using NeuroScan Acquire 4 at a 20 kHz analog-to-digital sampling rate. To keep the participant still but awake during electrophysiological testing, the participant watched a movie of his or her choice in a comfortable reclining chair. The left ear remained unoccluded during the recording session so that the movie soundtrack was audible. The stimulus was presented in the context of multi-talker background babble. The stimulus was presented at a signal-to-noise ratio of -10 dB relative to the root mean square amplitude of the background noise.

Although normal language processing generally involves the use of attention, it also relies upon other, automatic processes. The ability to consciously perceive the meaning of speech presented in noise, for example, depends upon the ability to accurately, efficiently, and precisely represent acoustic characteristics of sound. The automatic representation of the basic characteristics of sound can be captured in the auditory brainstem response, which can be elicited when a subject is performing a task unrelated to the target stimulus or is asleep (Skoe and Kraus, 2010). Despite the passive nature of the recording paradigm, characteristics of the auditory brainstem response such as the strength of spectral encoding and the timing of identifiable peaks in the waveform have been linked to abilities such as reading (Anderson et al., 2010), speech in noise perception (Kraus and Chandrasekaran, 2010), and consonant-vowel syllable discrimination in noise (de Boer et al., 2012). The fact that the auditory brainstem response can be elicited even if attention is not directed to the target stimulus is a major strength of the methodology, allowing researchers to assess auditory encoding with a technique relatively unaffected by transient changes in cognitive or emotional state. As a result, the auditory brainstem response has a high degree of test-retest reliability (Russo et al., 2004; Song et al., 2011), providing a stable snapshot of an individual’s auditory encoding. Another important characteristic is that, due to temporal precision of subcortical nuclei and their ability to phase-lock to relatively high frequencies (up to 1,000 Hz; Liu et al., 2006), the response mirrors many of the acoustic characteristics of the evoking stimulus (Galbraith et al., 1995). In contrast, the cortical response can only phase-lock up to frequencies of roughly 100 Hz (Steinschneider et al., 2008), and as a result cortical responses do not actively reproduce spectrotemporal content in the frequency range of speech formants.

Electrophysiological responses were bandpass filtered oﬄine in Neuroscan Edit (Compumedics) from 70 to 2000 Hz (12 dB/octave, zero phase-shift) to include energy within the phase-locking limits of the midbrain (Liu et al., 2006) and to minimize low-frequency cortical activity. Responses were pre-stimulus baseline corrected and epoched over a -40 to 190 ms window, with stimulus onset occurring at time 0. An artifact reject criterion of ±35 μV was applied. A final added response representing 6000 trials, 3000 from each stimulus polarity, resulted for each subject.

### DATA ANALYSES

To investigate timing shifts between pre- and post-training, we employed two methods: stimulus-to-response correlation (Skoe and Kraus, 2010) and the cross-phaseogram. Responses at pre- and post-training sessions were compared to the original stimulus by identifying the shift that was necessary to maximize the cross-correlation between the response to the stimulus, with this shift limited to values between 7 and 14 ms. This procedure identifies the neural transmission delay (or “lag”) between presentation of a stimulus and the neural response.

The cross-phaseogram (Skoe et al., 2011) is an objective measure of timing that relates strongly to timing shifts of response peaks (Tierney et al., 2011). A cross-phaseogram was constructed for each subject, using custom routines coded in MATLAB (The MathWorks Inc.): phase shifts were calculated on 40 ms overlapping windows of the response; the midpoint of the first window started at 10 ms, with each subsequent window shifted by 1 ms, and the final window centered on 70 ms. First, each of these windows was baseline-corrected, then ramped on and off using a Hanning window. Next, the cross-frequency spectrum of each window was calculated and converted to phase angles using the cross-power spectral density function. Jumps between successive blocks of greater than π were corrected to their 2π complement. The resulting cross-phaseogram plot is a three-dimensional (3D) image, with the degree of shift mapped to different values on the red-green-blue color spectrum. Regions colored in green indicate that there was no effect of training on the phase of responses. For regions appearing red, the response at post-training was earlier relative to responses to pre-training; for regions colored in blue, responses post-training were later than responses to (da) pre-training. Average phase shifts over 70–400 Hz during the 10–70 ms dynamically changing portion of the response were analyzed between groups. This frequency band was previously shown to be important in identifying differences in encoding (da) presented in quiet and noise (Tierney et al., 2011).

## RESULTS

**FIGURE 2:**
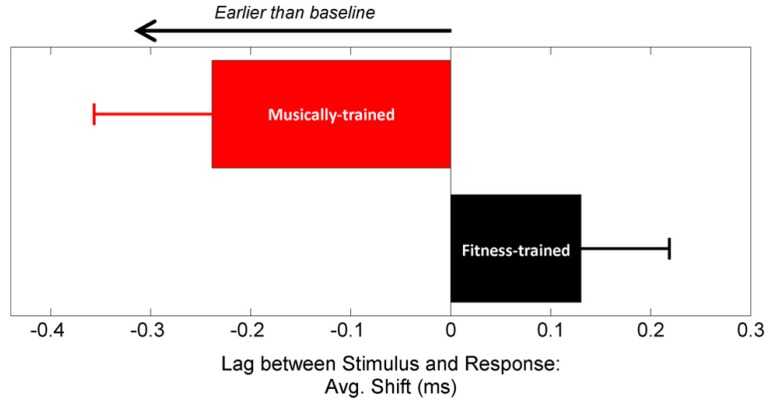
**In-school music training increases neural resilience to the effects of noise.** After 2 years, the stimulus-to-response lag was earlier for the musically trained group (red) while the fitness-trained group (black) did not change from pre-test. Stimulus-to-response lag was computed automatically using a cross-correlation algorithm. Error bars represent ±1 standard error of the mean.

**FIGURE 3:**
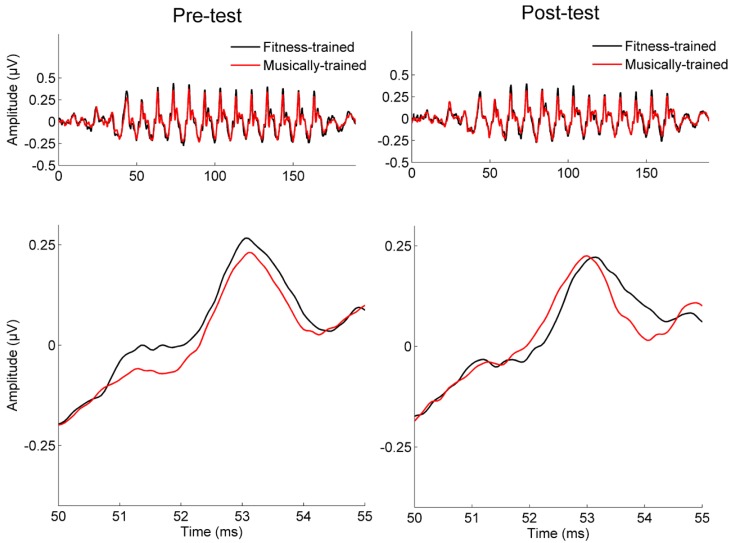
**Pre-test and post-test waveforms in musically trained and fitness-trained groups.** Grand average neural responses for the musically trained (red) and fitness-trained (black) groups at pre-test and post-test, displayed across the entire subcortical response (top) and at a single response peak (bottom).

Neural response timing was analyzed using two converging methods. First, we measured the lag between the stimulus and response using cross-correlation (Skoe and Kraus, 2010), with a greater lag in neural response timing reflecting greater neural delays (**Figure 2**). Using a repeated measures ANOVA with testing year as the within-subject factor and training group as the between-subject factor, we found a significant interaction between year and training group [*F*(1,41) = 6.39, *p* = 0.015], but no main effects [Training group: *F*(1,41) = 0.155, *p* = 0.696; Year: *F*(1,41) = 0.553, *p* = 0.461]. One-tailed *post hoc* paired *t*-tests revealed that between years, stimulus-response lag decreased for the musically trained group [shift = -0.25 (0.56) ms; *t*-stat = 2.03, *p* = 0.028] but not the fitness-trained group [shift = 0.14 (0.43) ms; *t*-stat = -1.48, *p* = 0.923]. The two groups were matched on stimulus-response lag in year 1 (*t*-stat = 1.19, *p* = 0.239], confirming that the different effects of training were not driven by pre-existing differences in neural timing. See **Figure 3** for a depiction of average waveforms in the two training groups at pre-test and post-test.

**FIGURE 4:**
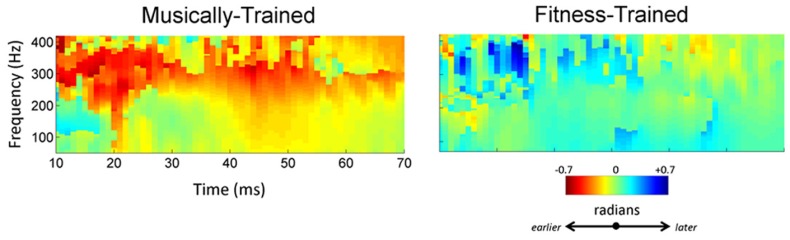
**High school music classes lead to earlier brain responses to speech.** Following training, the music group (left) shows an earlier response as evidenced by a negative phase-shift within the 70–400 Hz range that appears as a band of red. The neural response of the fitness training group (right) was stable (green) from pre- to post-test.

To confirm the effect of musical training, we computed phase shifts between responses collected before and after 2 years of training (**Figure 4**). This method generates a measure of timing shift between two recordings that correlates with shifts in manually marked peak latencies (Tierney et al., 2011). Following training, musician responses were earlier [-0.20 (0.40) radians], while the response of the fitness-trained participants remained unchanged [0.11 (0.42) radians]. These two shifts were significantly different (*t*-stat = 2.51, *p* = 0.0016). One-tailed *t*-tests revealed that the music group’s shift (*t*-stat = 2.34, *p* = 0.0149), but not the fitness group’s shift (*t*-stat = 1.24, *p* = 0.887) was significantly smaller than zero, indicating that enhancements in the timing of neural responses to noisy speech were exclusive to music training.

## DISCUSSION

Here we show that high school music instruction enhances the neural representation of speech in background noise, a neural advantage previously found to result from more extensive one-on-one training. Moreover, our subjects live in relatively low-income areas and reported relatively low levels of socioeconomic status (SES). Given that SES impacts language functioning (Hackman et al., 2010) and the neural encoding of speech (Skoe et al., 2013), our results suggest that affordable in-class musical training may be able to ameliorate some of the negative consequences of impoverishment.

Some musician advantages are larger if training is begun earlier in life (Penhune, 2011), and so the effects of in-class music training may be even larger in younger populations. Nevertheless, we find that 2 years of in-class training in adolescence can enhance how the brain encodes speech. Though neural plasticity has declined somewhat by the time a child reaches adolescence, the window for successful training-based intervention remains open. As computer-based training can enhance sensory processing even in older adult subjects (Mahncke et al., 2007; Berry et al., 2010; Anderson et al., 2013), it may never be too late to benefit from newly acquired experience such as music instruction.

Much of the research on musical training’s effects on the brain has compared subjects with many years of extensive musical training to those without. These group differences are then assumed to result from musical experience. However, it is possible that individuals with superior auditory abilities are more strongly drawn to music as a hobby or career. Although correlations between extent of musical experience and neural function (Parbery-Clark et al., 2009a; Strait et al., 2012; reviewed in Strait and Kraus, 2013) support training-dependent plasticity, it remains possible that subjects with certain characteristics, whether environmental or genetic, are more likely to continue their training rather than abandoning it. Here, by using a longitudinal approach to examine neural changes in students who were matched in reading, IQ, and neural function before training began, we present the strongest evidence to date for a causative role of in-school musical training in modulating the neural encoding of speech.

In the musically-trained group, the neural responses were found to be 0.25 ms earlier after two years of training. Although 0.25 ms is a small difference in latency compared to the duration of a word or a sentence, auditory brainstem response latency differences of as little as 0.2 ms are clinically significant. For example, small differences in the timing of brainstem responses elicited by presentation to each ear can be used to diagnose the presence of tumors of the vestibulocochlear nerve (Grayeli et al., 2008). The consistent associations found between auditory brainstem latency and language skills such as speech-in-noise perception (Kraus and Chandrasekaran, 2010), reading (Anderson et al., 2010), and consonant-vowel discrimination in noise (de Boer et al., 2012) suggest that early brainstem timing is crucial for auditory processing. Moreover, the reversal of age-induced delays in neural timing by auditory training (Anderson et al., 2013) suggests that earlier neural timing is advantageous. The exact mechanisms by which auditory brainstem latency influences auditory processing, however, remain a subject for future research. The enhancements reported here, therefore, suggest that our musically trained participants benefit from improved speech in noise perception and reading abilities. Classrooms are not ideal acoustic environments for instruction: background noise commonly exceeds recommended levels (Knecht et al., 2002) and higher levels of background noise are linked to worse performance on standardized tests (Shield and Dockrell, 2008). Perception of speech in noise, therefore, may be vital for a child’s ability to understand what is being communicated in classrooms. Therefore, our finding of an enhancement of the neural encoding of speech in noise, along with previously reported cognitive benefits of long-term musical training (reviewed in Strait and Kraus, 2013), suggest that musical training may be able to improve academic performance by training perceptual and cognitive skills (such as auditory working memory, reading, and speech in noise perception) on which scholastic ability depends. Future work should examine the effects of music classes on scholastic measures such as standardized tests or grades and investigate whether any academic enhancements due to music training can be attributed to increased perceptual or cognitive skills. As a result, we suggest that, when considering the role of music education in school, its potential linguistic, cognitive, and scholastic benefits should be factored in alongside its more obvious esthetic benefits. Future work should investigate how these neural changes translate to academic benefits, as well as whether training-induced enhancements persist after instruction ceases (Skoe and Kraus, 2012; White-Schwoch et al., 2013). Another important direction for future work concerns the delineation of the different sub-components of musical training responsible for certain neural enhancements. For example, music reading, ear training, group synchronization, and solo practice may all have different effects on the developing brain. Yet another potentially fruitful direction for future research is in identifying functional and structural features of the brain that predict the ability to benefit from music education (Zatorre, 2013).

It remains an open question how the benefits of music training for auditory neural encoding compare to more language-directed computer-based auditory training or one-on-one speech therapy. Benefits for speech-in-noise processing may be achievable through other means besides music. In practice, however, it is difficult to ensure steady engagement with an auditory training program for extended periods of time, because waning motivation leads to decreased participant compliance and because such programs are often not designed to be used for lengthy periods. Music’s inherently rewarding and emotionally evocative nature (Patel, 2011; Salimpoor et al., 2013), on the other hand, make it a uniquely sustaining way to train auditory skills.

One-on-one speech therapy could be a more feasible way to train speech listening skills for a sustained period of time, as the personal interaction included as part of the therapy would likely be more engaging for the participant, leading to greater long-term compliance. Speech therapy is comparatively expensive, however, requiring the personal attention of a trained therapist, while the enhancements that we demonstrate are the result of classroom-based music training. Future work should directly test the comparative value provided by in-school music training versus speech therapy in terms of benefits versus costs. Furthermore, both speech therapy and computer-based auditory training remove children from the classroom, while music classes take place within the school curriculum as part of the regular school day. Future work should directly test the comparative value provided by in-school music training versus speech therapy in terms of benefits versus costs. The benefits of music training also extend beyond speech processing, encompassing cognitive benefits such as auditory attention and working memory (reviewed in Kraus et al., 2012). Ultimately, music training and speech therapy are not mutually exclusive options; the largest benefit would likely be gained by students who engage in both kids of training.

In summary, in-school group training during adolescence can enhance the brain’s processing of speech in noise. As such, the enhancement of speech encoding by musical experience may not require the development of expert musical skills, and is accessible regardless of age or income. This study is consistent with the notion that music is an important part of a well-rounded school curriculum, alongside foreign language instruction, math, reading, and other elements vital for a child’s development.

## Conflict of Interest Statement

The authors declare that the research was conducted in the absence of any commercial or financial relationships that could be construed as a potential conflict of interest.
